# Novel Agmatine Derivatives in *Maerua edulis* With Bioactivity Against *Callosobruchus maculatus*, a Cosmopolitan Storage Insect Pest

**DOI:** 10.3389/fpls.2018.01506

**Published:** 2018-10-17

**Authors:** Philip C. Stevenson, Paul W. C. Green, Iain W. Farrell, Alice Brankin, Brighton M. Mvumi, Steven R. Belmain

**Affiliations:** ^1^Natural Resources Institute, University of Greenwich, Chatham, United Kingdom; ^2^Royal Botanic Gardens, London, United Kingdom; ^3^Jealott’s Hill International Research Centre, Syngenta, Bracknell, United Kingdom; ^4^Department of Soil Science and Agricultural Engineering, University of Zimbabwe, Harare, Zimbabwe

**Keywords:** botanical insecticide, cowpea weevil, pesticidal plant, postharvest pest management, chemical and structural analyses

## Abstract

Food security in developing countries is threatened by crop pests and ectoparasites in livestock. Strategies for their management still rely on synthetic pesticides which are not always effective and the active ingredients persist in the environment with negative consequences for beneficial arthropods, farmers and consumers, hence necessitating research on sustainable alternatives. Botanical insecticides are increasingly relevant, typically having lower impacts on users, consumers and the environment. One example is the southern African shrub the Blue bush-berry, *Maerua edulis*. Recent work reported effective pest control using this plant species against cattle ticks, storage beetles and vegetable pests. However, little is known about the chemistry underlying activity and this is essential to optimize its use. Here, we identified two novel plant chemical structures, the *E* and *Z* isomers of cinnamoyl-4-aminobutylguanidine along with the *E* and *Z* isomers of 4-hydroxycinnamoyl-4-aminobutylguanidine in the leaves of *M. edulis*. We isolated these compounds from the leaves and elucidated their chemical structures using various spectroscopic techniques including High Resolution Mass Spectrometry and Nuclear Magnetic Resonance Spectroscopy. We also identified a further 11 closely related structures of which 6 are tentatively reported here for the first time. Stachydrine and 3-hydroxystachydrine were also identified in the leaf extract, and occurred at very high concentrations; up to 2% w/w of dry leaves. We tested these two compounds, along with the 4 main cinnamoylamides and the crude *M. edulis* leaf extract against the cowpea bruchid *Callosobruchus maculatus* at concentrations equivalent to those present in extracts used by smallholder farmers. Mortality of insects exposed to crude plant extracts after 72 h was significantly higher than the untreated control although still lower than for insects exposed to rotenone, the positive control. The two new compounds and stachydrine showed similar activity to the crude extracts suggesting that these compounds explained the activity of the extract. After 6 days, the mortality of insects exposed to crude extracts and isolated compounds was similar to that recorded with the positive control. The stachydrine fraction and the *E* and *Z* isomers of cinnamoyl-4-aminobutylguanidine also inhibited oviposition activity in fecund female beetles. Our data show that methanol extracts of *M. edulis* were toxic to *C. maculatus* and inhibited oviposition even at 0.1% w/v so these foliar chemicals may explain the activity of the plant material. We also synthesized the amides which facilitated structural elucidation, produced adequate quantities for testing and demonstrated the potential for commercial synthesis.

## Introduction

Plant-based pesticides contribute a fraction of that provided by commercial pesticides in industrialized nations (Isman, 2006) yet remain the major pest control technology in less developed regions such as Africa where they are used as dry admixes or crude extracts (Kamanula et al., 2010; Nyirenda et al., 2011; Stevenson et al., 2017). While small holder farming systems are where pesticidal plants have the greatest potential (Isman, 2008), their acceptance as commercial alternatives to synthetic products is growing (Isman, 2015). This is especially pertinent in the light of regulatory changes to synthetic pesticide use globally (Isman, 2017). Despite the widespread use of pesticidal plants surprisingly little is known about the chemical mechanisms underlying activity in most species. This limits opportunities to maximize efficacy. For example, information about the bioactive chemicals would allow the selection of elite provenances of plants that produced consistently high levels of active ingredients (Sarasan et al., 2011) while application protocols could be optimized based on a knowledge of the solubility of active ingredients; their concentrations in plant material grown under different conditions (Belmain et al., 2012; Stevenson et al., 2012) or their stability in UV light (Angioni et al., 2005; Caboni et al., 2006). For example, field efficacy of pesticidal plant materials for which the bioactivity is associated with lipophilic compounds is optimized by extraction in weak detergents (Mkenda et al., 2015; Mkindi et al., 2017).

*Maerua edulis* (Gilg & Gilg-Ben.) DeWolf (Capparaceae) is a shrub widely distributed in Southern Africa where it has reported antimicrobial and pesticidal activity (Nyahangare et al., 2012, 2017; Mazhawidza and Mvumi, 2017). To date, however, nothing is known about the components that mediate these reported activities limiting the potential to maximize the pest control benefits of *M. edulis* for smallholder farmers or understand more about the potential toxicity of the genus (Malami et al., 2014; Nyahangare et al., 2016). There are currently a further 13 species of *Maerua* in southern Africa, (Swanepoel, 2015) so there may be others that have use as botanical pesticides. The chemistry of some of these other species has been studied. For example, lupane triterpenoids were isolated from *Maerua oblongifolia* although no biological activity of these components was established (Abdel-Mogib, 1999) while lipids and triterpenes were isolated from *M. crassifolia* (Ibraheim et al., 2008). Some previous work has also studied the water clarifying properties of *Maerua* species and its antibacterial properties and attempted to identify some of the compounds that might explain this activity, but without success (Luo et al., 2011a). Elsewhere some work has reported the occurrence of several fatty acids, sterols and phenolic acids (Ali et al., 2008).

Here, we report the occurrence of two novel and two known cinnamoylagmatine derivatives along with stachydrine and hydroxystachydrine in the leaves of *M. edulis* and identify up to 6 further new compounds based on High Resolution Electrospray Ionization Mass Spectrometry (HR-ESI-MS). We describe the full synthesis and structural analysis of the agmatine derivatives and evaluate their biological activity against a target storage pest insect of beans explaining the reported effectiveness of this plant in pest control by smallholder farmers.

## Materials and Methods

### Chemical Analysis

Extracts of *M. edulis* (50 mg ml^-1^, in methanol for 24 h) were analyzed by LC-ESIMS using an LTQ Orbitrap XL, linear ion trap/orbitrap hybrid mass spectrometer (Thermo Scientific, San Jose, CA, United States) with an electrospray ionization source (Ion Max, Thermo Scientific) coupled to an Acella 1250 uPLC system (Thermo Scientific). Samples were injected onto a Phenomenex Luna C_18_(2) column (150 × 3 mm i.d., 3 μm particle size) and eluted at 400 μL min^-1^ using a linear gradient as described previously (Green et al., 2017) and compounds eluted using MeOH (A), H_2_O (B), and formic Acid (C) with *A* = 0%, *B* = 90% at *T* = 0 min; *A* = 90%, *B* = 0% at *T* = 20 min and held for 10 min with C at 10% throughout the analyses. Column temperature was 30°C with flow rate = 0.5 ml min^-1^. Samples were scanned, using FTMS, from *m/z* 125–1250 in both positive and negative modes.

### Isolation of Agmatine Derivatives and Stachydrine

Dried leaves (100 g) were finely ground in a coffee grinder (Krups F203) and extracted with aqueous methanol (50%, 500 ml) for 24 h. After filtering and concentrating to dryness, the solid residue was redissolved in 5% MeOH and fractionated using reverse phase vacuum column chromatography (ISOLUTE Flash C18 under vacuum) into three fractions: 2% methanol containing compounds **5** and **6**; 10% methanol containing **1** and **2** and a 20% methanol fraction containing **3** and **4**.

An HPLC system consisting of a Waters 2695 separations module linked to a 2996 photodiode array detector (PDAD) was used for further isolation of the compounds from extracts. Aliquots of *M. edulis* extract (200 μL) were injected onto a Phenomenex Luna RP18 column (300 × 10 mm, length × i.d.; 10 μm particle size) and eluted at 2 mL min^-1^ using a linear gradient with *A* = 95%, *B* = 5% at *T* = 0; *A* = 0%, *B* = 5%, *C* = 95% at *T* = 20 min) where *A* = water; *B* = 1% formic acid in acetonitrile and *C* = MeCN. HPLC isolation yielded 1.6, 0.7, 3.3, and 10.0 mg of **1**–**4**, and samples were subjected to NMR. NMR spectra were acquired in MeOH-*d*4 at 30°C on a Bruker Avance 400 MHz instrument. Standard pulse sequences and parameters were used to obtain one-dimensional ^1^H, ^13^C spectra and two-dimensional gradient-enhanced COSY, HSQC, and HMBC spectra. Chemical shift referencing was carried out using the internal solvent resonances at *d*H 3.31 and *d*C 49.1 (calibrated to TMS at 0.00 ppm). Compounds **5** and **6** could not be separated using this method but were isolated together as fraction containing both compounds in a proportion of 1:2. Both were identified by interpretation of the HR-ESI-MS data and comparison with both 3-hydroxystachydrine (**5**) and stachydrine (**6**) from our in house MSn library and were consistent with published data (Cornforth and Henry, 1952; Xie et al., 2015). All compounds were quantified against standard curves from isolated compounds or authentic standards.

### Synthesis of E-Cinnamoyl Agmatines and Their Isomerisation

Agmatine sulfate (500 mg, 2.2 mmol, Sigma-Aldrich, > 97%) was dissolved in 2.5 ml deionized water. Sodium hydroxide (175 mg, 4.4 mmol, Sigma-Aldrich, 98.6%) was dissolved separately in 2.5 ml deionized water and allowed to cool back to room temperature (ca. 20°C). Half of the aqueous sodium hydroxide was added to the agmatine sulfate solution followed by powered cinnamoyl chloride (366 mg, 2.2 mmol, Sigma-Aldrich, 98%) and stirred with a magnetic ‘flea’. The pH was maintained at around 9 to 10 by further dropwise addition of aqueous sodium hydroxide and when no further change in pH occurred, indicating complete reaction, the mixture was left stirring for an hour. The product was isolated by reverse phase vacuum column chromatography as described above and eluted in the 20% aqueous methanol fraction. This yielded 44 mg of *E*-cinnamoylagmatine (8% yield). Unchanged agmatine was recovered from a 2% aqueous methanol fraction, dried down and re-cycled through the synthesis. An aliquot (20 mg = 0.077 mmol) of the *E*-isomer was dissolved in 2 ml aqueous methanol and exposed to UV light (254 nm) for 72 h. The resulting mixture contained *Z* and *E*-isomers in the proportion 1:2 which were separated using HPLC as described above. Both the *E* and *Z* synthetic isomers were found to be identical with the compounds isolated from the dried plant by HR-ESI-MS and NMR.

### Insects

*Callosobruchus maculatus* were a Ghanaian strain, originally collected from the wild in 1995 (Kestenholz et al., 2007) and reared at Kew for 20 generations under laboratory conditions. They were kept in 1 L Kilner jars with perforated lids and housed in a temperature-controlled room (28 ± 1°C) that was kept in permanent darkness. Humidity was ambient, within the range 55–60%. Gravid females laid eggs on cowpea seeds *Vigna unguiculata* L. during 3 days of exposure to un-infested seeds. Adults were then removed and 24–28 days later the next generation of adults emerged. The insects used for bioassays were 3–5 days post-emergence.

### Plant Material and Extraction

*Maerua edulis* plants were collected from Chiredzi district about 430 km East of Harare in Zimbabwe and identified locally as previously reported (Nyahangare et al., 2017). A specimen is deposited in the national herbarium and botanic gardens of Zimbabwe (Voucher specimen recorded as Nyahangare E5).

### Bioassays of Extracts and Synthesized Compounds

Dried and powdered *M. edulis* leaf was extracted for 24 h in methanol (10% w/v). This was filtered, and aliquots diluted to 1 and 0.1% by weight of plant material for bioassays and chemical analysis. Isolated compounds and the stachydrine fraction containing a 1:2 mixture of **5** and **6** were dissolved to concentrations representing their proportions in 10, 1 and 0.1% w/v extracts of *M. edulis* leaf. Specifically, these were 250, 25 and 2.5 ppm (**1**); 170, 17 and 1.7 ppm (**2**); 750, 75 and 7.5 ppm (**3**); 260, 26 and 2.6 ppm (**4**) and 3200, 320 and 32 ppm for stachydrine fraction (**5/6**). Following a protocol from established methods for testing the biological activity of plant compounds against bruchids (Stevenson et al., 2016) aliquots (70 μL) of compounds or extracts were evaporated onto vials (25 mL, nominal capacity) under a stream of air and with constant rotation of the vial. To determine the toxicity of extracts and pure compounds between 5 and 12 insects were added to each experimental vial, ensuring that the ratio of males to females was at least equal to minimize competition between males, with each treatment replicated 10 times. Thus each treatment was tested against up to 120 insects. Insects could walk freely within the vial and thus encounter the compounds through contact. After 72 h mortality was assessed.

Cowpea beans (*V. unguiculata*) (black-eyed peas) (*N* = 5) were then added to each vial after 72 h. This permitted an assessment of oviposition among surviving insects. After a further 72 h mortality was recorded once more. The numbers of eggs laid on both the vials and the beans were counted and from these data the eggs laid per female was calculated. Differences among treatments in insect mortality and eggs laid were assessed for significance by analysis of variance (ANOVA) and Tukey’s *post hoc* honestly significant difference (HSD) test to separate the means at the 95% confidence interval. Analyses were performed in XLSTAT version 2015.1.01 (Addinsoft, Paris, France). Datasets are available on request.

## Results

### Chemical Analysis

Tentative molecular formulae were assigned to 6 compounds. Compounds **1–4** were isolated as off-white solids and characterized initially by UV spectra that were similar to coumaric and cinnamic acid but with molecular formulae indicating that they occurred as higher molecular weight derivatives. Compound **1** and **2** had a UV (MeOH) λ_max_ nm: 224, 293, and 269 respectively; HR-ESI-MS *m/z*: 277.1664 and 277.1658 [M+H]^+^ respectively, calcd. for C_14_H_21_N_4_O_2_^+^: 277.1659. Compound **3** and **4** had a UV (MeOH) λ_max_ nm: 276 and 252 respectively; HR-ESI-MS *m/z*: 261.1712 [M+H]^+^ calcd. for C_14_H_21_N_4_O^+^: 216.1710.

**FIGURE 1:**
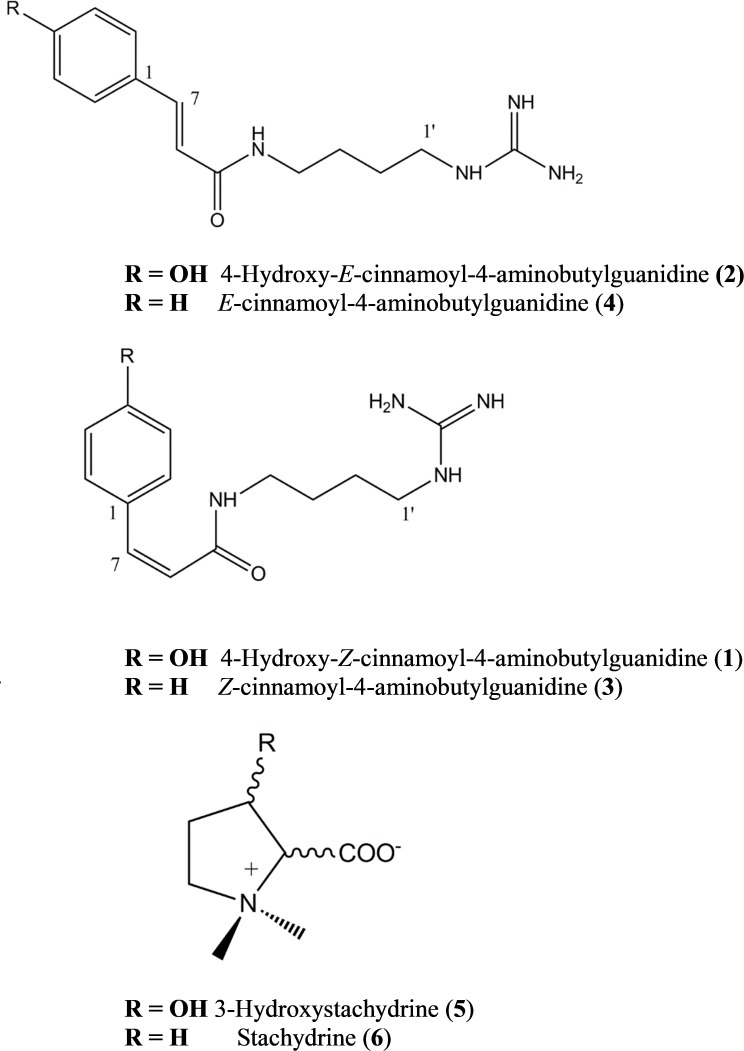
*Maerua edulis* metabolites isolated from leaves and synthesized (**1** to **4** only).

Compound **1** was identified as 4-hydroxy-*Z*-cinnamoyl-4-aminobutylguanidine (*p*-coumaroylagmatine) by comparison of its ^1^H NMR spectrum with published data for this compound (Ueda et al., 1998) although the *trans* (*E* form) isomer was isolated earlier (Stoessl, 1965) (**Figure 1**). Based on correlations of the α (H-8) and β (H-7) olefinic protons in the HMBC experiments here we suggest that the orientation of these protons in Ueda et al. (1998) was incorrect and should be H-7, δ_H_ 6.80 *d* (*J* = 12.3 Hz) and H-8, δ_H_ 5.96 *d* (*J* = 12.3 Hz). The main difference in the ^1^H NMR spectrum of **2** compared with **1** was a downfield shift of the resonances for H-7 and H-8 to δ_H_ 7.42 and δ_H_ 6.43, respectively, along with the magnitude of the coupling at *J* = 15.8 typical for cinnamoyl derivatives in the *E* configuration (Stevenson et al., 1993). Thus **2** was determined as 4-hydroxy-*E*-cinnamoyl-4-aminobutylguanidine (*E*-coumaroylagmatine). A full data set of NMR resonances for **2** are presented in **Table 1**.

**Table 1:** ^1^H and ^13^C NMR spectroscopic data (*δ* ^1^H, *J* in Hz) for **2–4** in D_2_O.

	2		3		4	
			
Atom	C δ ppm	H δ ppm	C δ ppm	H δ ppm	C δ ppm	H δ ppm
1	127.5		135.0		136.0	
2	130.5	7.50 *d* (8.6)	128.5	7.60 *m*	129.0	7.39 *m*
3	116.5	6.91 *d* (8.6)	129.7	7.46 *m*	129.1	7.38 *m*
4	158.1		130.8	7.45 *m*	129.2	7.38 *m*
5	116.5	6.91 *d* (8.6)	129.7	7.46 *m*	129.1	7.38 *m*
6	130.5	7.50 *d* (8.6)	128.5	7.60 *m*	129.0	7.39 *m*
7	141.3	7.42 *d* (15.8)	141.5	7.48 *d* (12.4)	137.3	6.91 *d* (15.8)
8	118.2	6.43 *d* (15.8)	120.8	6.58 *d* (12.4)	124.4	6.09 *d* (15.8)
9	169.7		169.3		171.2	
1′	41.4	3.20 *t* (6.3)	41.4	3.19 t	41.4	3.09 *t* (6.9)
2′	25.9	1.61 *m*	25.9	1.60 *m*	26.0	1.37 *m*
3′	26.3	1.60 *m*	26.3	1.60 *m*	25.7	1.45
4′	39.5	3.29 *t* (1.61)	39.6	3.30 *m*	39.3	3.18
guanine	157.4		157.4		157.3	


Molecular formulae for compounds **3** and **4** indicated one fewer oxygen compared to **1** and **2**. The ^13^C spectrum of **3** and **4** also recorded only 12 resonances instead of the expected 14 owing to symmetry in the phenyl ring with C-2 and C-6 and C-3 and C-5 recording identical shifts. A resonance at 171.16 ppm in the ^1^H spectrum was consistent with an amide carbonyl and a quaternary carbon at 157.26 was as expected for the guanidine carbon (158.7) (Ueda et al., 1998; Huang and Lee, 2005). A group of five protons in the region 7.36–7.40 with three similar carbon shifts in the range 129.01–129.16 indicated an unsubstituted phenyl group typical of cinnamic acid. A pair of doublets (*J* = 12.4 Hz) at δ_H_ 6.91 and δ_H_ 6.09 corresponded with the olefinic protons α (H-8) and β (H-7) in **1** indicating a cinnamoyl moiety in the *Z*-configuration for **3.** The strong coupling from δ_H_ 6.91 in **3** in the HMBC with C-2/6 indicated a stronger correlation with the phenyl carbons and hence was β- to the amide carbonyl while a weak correlation from δ_H_ 6.09 with C-2/6 indicated this proton was H-8 was α (**Table 1**). Other resonances in the agmatine moiety were consistent with published data (Huang and Lee, 2005). NMR resonances from **4** were similar to **3** differing only in the down field shift of the olefinic protons to δ_H_ 7.48 and H-8 δ_H_ 6.58 and an increase in magnitude of the coupling to *J* = 15.8 Hz typical for a cinnamoyl moiety in the *E* configuration (**Table 1**).

Compounds **5** and **6** were identified by interpretation of HR-ESI-MS data and comparison with in house libraries for both 3-hydroxystachydrine and stachydrine. In HR-ESI-MS compound **5** gave *m/z*: 160.0972 [M+H]^+^ calcd. for C_7_H_15_NO_3_^+^ while compounds **6** gave *m/z*: 144.1021 [M+H]^+^ calcd. for C_7_H_15_NO_2_^+^: and were consistent with published data (Cornforth and Henry, 1952; Xie et al., 2015).

The occurrence of hydroxycinnamoylagmatines is restricted to only a few species but are best described from *Hordeum vulgare* (Barley) where they occur as precursors to antifungal hordatines in a wide variety of forms although none as the cinnamoyl derivatives found here in *M. edulis* (Gorzolka et al., 2014; Heuberger et al., 2014; Pihlava, 2014). Similarly, here, evidence from HR-ESI-MS data allowed tentative identification of numerous other closely related 4-aminobutylguanidine derivatives (**Table 2**) and indicated that these structures are highly diverse and so may be important to their ecological function. The occurrence of several additional related compounds that may have similar biological activities provides scope for further study of other active components in *M. edulis* leaves. We did not identify the various hordatines often identified together with cinnamoylagmatines (Pihlava, 2014).

**Table 2:** Assignments of minor cinnamoylagmatines and their sugar conjugates in *M. edulis* based on HR-ESI-MS data.

Assignment	Rt (min)	Observed [M+H]^+^ m/z	Calc. [M+H]^+^ m/z	Error (ppm)	Elemental composition
Hydroxycoumaroylagmatine	3.22	293.1620	293.1614	1.1	C_14_H_20_N_4_O_3_
Methoxycinnamoylagmatine^∗^	7.96	291.1831	291.1821	1.6	C_15_H_22_N_4_O_2_
Feruloylagmatine^∗^	5.63	307.1769	307.1770	0.17	C_15_H_22_N_4_O_3_
Hydroxyferuloyl agmatine^∗^	3.50	323.1727	323.1719	1.3	C_15_H_22_N_4_O_3_
N1-(3,4-Dimethoxy-*E*-cinnamoylagmatine)^∗^	7.61	321.1938	321.1927	1.1	C_16_H_24_N_4_O_3_
Sinapoylagmatine^∗^	5.92	337.1877	337.1876	0.6	C_16_H_24_N_4_O_3_
Cinnamoylagmatine hexoside^∗^	4.05	439.2209	451.2187	3.1	C_20_H_30_N_4_O_7_
Methylcoumaroylagmatine hexoside	7.20	453.2351	453.2349	0.8	C_21_H_32_N_4_O_7_
Feruloylagmatine hexoside	2.77	469.2316	469.2298	2.3	C_21_H_32_N_4_O_8_
Methylferuloylagmatine hexoside	5.27	483.2477	483.2455	2.8	C_22_H_34_N_4_O_8_
Sinapoylagmatine hexoside	3.94	499.2423	499.2404	2.5	C_22_H_34_N_4_O_9_


*Some compounds have been reported previously (Gorzolka et al., 2014; Heuberger et al., 2014; Pihlava, 2014) but the remainder indicated by ^∗^ tentatively represent novel compounds.*

### Bioactivity

To provide sufficient quantities of these compounds to test against our target insect we synthesized compounds **2** and **4** by combing agmatine sulfate and the *E*-cinnamoyl chloride or hydroxycinnamoyl chloride as described above and the *Z* isomers of each (**1** and **3**) were produced by irradiation of **2** and **4** with UV light at 254 nm for 72 h followed by separation on semi-preparative HPLC.

We evaluated the biological activity of a crude extract of *M. edulis*, along with compounds **1–4** and stachydrine fraction (**5/6**) against *C. maculatus* at concentrations equivalent to those present in a 10%, 1, and 0.1% crude leaf extract, which was in the range of concentrations used by farmers (Nyahangare et al., 2017). After 72 h there were differences in mortality (ANOVA, 19 d.f., *P <* 0.001) with more of those insects exposed to the crude extract dying than the control group (Tukey’s, *P* < 0.05) although this was still lower than for insects exposed to the positive control, rotenone (Tukey’s, *P* < 0.05; **Table 3**). Compounds **3** and **4** and the stachydrine fraction showed mortality effects that were similar to the crude extracts (Tukey’s, *P >* 0.05) suggesting that these compounds contribute significantly to the activity of the extract after 72 h. However, fewer insects were affected than for those exposed to the positive control rotenone (Tukey’s, *P <* 0.05). Although there were differences in mortality after 144 h (ANOVA, 19 d.f., *P <* 0.001; **Table 3**), the mortality of insects on the crude extracts, compounds **1–4** and stachydrine fraction (**5/6**) were similar to that recorded with rotenone the positive control (Tukey’s, *P >* 0.05). Egg laying was affected by the different treatments (ANOVA, 19 d.f., *P <* 0.001; **Table 3**) with compounds **3, 4** and the stachydrine fraction (**5/6**) inhibiting egg laying activity in fecund female beetles. Our data show that methanol extracts of *M. edulis* were toxic to *C. maculatus* and inhibited oviposition even at 0.1% w/v so these foliar chemicals explain the activity of plant material.

**Table 3:** Treatment effect on percentage mortality of *Callosobruchus maculatus* after 72 and 144 h and the numbers of eggs laid per female when exposed to extracts and compounds isolated from *Maerua edulis*.

Treatment	Concentration	72 h mortality	144 h mortality	Eggs per female
*M. edulis* crude extract (%w/v)	0.1	22.42 ^B,C,D,E,F^	79.34 ^A,B,C,D^	17.05 ^B,C,D^
	1	25.58 ^B,C,D,E,F^	76.01 ^A,B,C,D^	19.35 ^A,B,C,D^
	10	38.82 ^B,C^	74.89 ^A,B,C,D^	16.60 ^B,C,D^
Compound **1** (ppm)	2.5	17.32 ^C,D,E,F^	63.58 ^A,B,C,D^	31.23 ^A^
	25	12.27 ^E,F^	61.33 ^A,B,C,D^	23.64 ^A,B,C,D^
	250	11.30 ^E,F^	56.47 ^A,B,C,D,E^	25.14 ^A,B,C,D^
Compound **2** (ppm)	1.7	29.42 ^B,C,D,E,F^	54.35 ^C,D,E^	15.99 ^B,C,D^
	17	13.86 ^D,E,F^	59.71 ^A,B,C,D^	28.04 ^A,B,C^
	170	12.92 ^E,F^	53.19 ^C,D,E^	28.46 ^A,B^
Compound **3** (ppm)	7.5	22.85 ^B,C,D,E,F^	56.37 ^A,B,C,D,E^	16.36 ^B,C,D^
	75	25.57 ^B,C,D,E,F^	40.45 ^D,E^	14.52 ^D^
	750	20.87 ^B,C,D,E,F^	51.21 ^C,D,E^	13.34 ^D^
Compound **4** (ppm)	2.6	34.16 ^B,C,D,E^	85.02 ^A,B,C^	18.34 ^A,B,C,D^
	26	40.79 ^B^	94.91 ^A^	15.33 ^B,C,D^
	260	24.68 ^B,C,D,E,F^	54.92 ^B,C,D,E^	14.33 ^D^
Fraction **5/6** (ppm)	32	36.42 ^B,C,D^	70.53 ^A,B,C,D^	13.53 ^D^
	320	22.22 ^B,C,D,E,F^	64.28 ^A,B,C,D^	13.92 ^D^
	3200	27.83 ^B,C,D,E,F^	63.27 ^A,B,C,D^	15.25 ^B,C,D^
Rotenone (ppm)	1000	73.62 ^A^	93.84 ^A,B^	14.89 ^C,D^
Untreated	Control	8.94 ^F^	17.21 ^E^	31.05 ^A^
CI		8.87	15.23	5.13
SE		4.50	7.72	2.60
F		10.41	5.45	5.64


*Crude extract of *M. edulis* leaf was tested at 10, 1.0 and 0.1% w/v while **1** (*Z*-coumaroylagmatine), **2** (*E*-coumaroylagmatine), **3** (*Z*-cinnamoylagmatine), **4** (*E*-cinnamoylagmatine) and the stachydrine fraction containing **5** (3-hydroxystachydrine) and **6** (stachydrine) were tested at their equivalent concentrations (ppm) found in the crude extract. Least squared difference means are shown, with 95% confidence interval (CI) and standard errors (SE) indicated at the base of each column. Values in the same column followed by the same letter are not significantly different from each other at the 95% confidence interval using Tukey’s *post hoc* honestly significant difference (HSD) test. *N* = 10 with up to 12 insects per replicate.*

## Discussion

We report the isolation and structural elucidation of two new and two previously described cinnamoylagmatine derivatives from the leaves of *M. edulis* a species of plant that is used as a botanical insecticide in southern Africa (Nyahangare et al., 2012, 2017; Mazhawidza and Mvumi, 2017). Although the *E*-coumaroylagmatine was reported previously this was from a synthesis and so is reported here for the first time from nature. Surprisingly, no other compounds that might typically be found in aqueous extracts of angiosperms such as flavonoid glycosides and phenolic acids were identified in the extracts. The secondary metabolite chemistry of *M. edulis* leaves is thus unusual since it comprises primarily stachydrine and phenylpropanoid derivatives of agmatine. Stachydrine is not previously reported from *Maerua* spp. but is known elsewhere in the Capparaceae having been isolated and identified from *Capparis tomentosa* Lam. (Cornforth and Henry, 1952) thus it is not surprising to find it in *Maerua*. Hydroxycinnamoylagmatines are encountered infrequently in nature and are restricted to only a few species. They are mostly described from *Hordeum vulgare* (Barley) where it was reported to have antifungal activity (Stoessl, 1965). Elsewhere cinnamoyl derivatives of agmatine are only know previously from *Albizia julibrissin* (Leguminosae) (Ueda et al., 1998) while the cinnamic acid amides reported from *M. edulis* in the present study have never previously been reported (Gorzolka et al., 2014; Heuberger et al., 2014; Pihlava, 2014). We also provide the first evidence for the biological activity of cinnamoylagmatines against insects and specifically, *C. maculatus*, a cosmopolitan storage pest beetle of cowpea. We also report similar activity for stachydrine and 3-hydroxystachydrine. Interestingly, extracts of *M. edulis* have also been reported to be biologically active against microorganisms and specifically *Mycobacterium bovis* and *M. tuberculosis* although the compounds identified in *M. edulis* in that study were unsaturated fatty acids which are unlikely to explain the activity reported for the extracts of this plant (Luo et al., 2011a,b). It would be valuable to ascertain whether the cinnamoylagmatine derivatives identified here or their metabolic products account for activity against mycobacterium species. Antimicrobial activity of compounds in barley is associated with hortadines which are polymers of the hydroxycinnamoylagmatines (Pihlava, 2014). Our analysis also reveals a wide variety of related derivatives of cinnamoylagmatines (**Table 2**) and by comparison with the tentative assignments published by Pihlava (2014) suggest up to 6 further novel compounds.

All compounds isolated from leaves of *M. edulis* and tested here (**1**–**6**) were biologically active against adult bruchid beetles and it is likely therefore that in the absence of other candidates in aqueous extracts, that these compounds explain the biological activity of the crude extracts as used by farmers and the activity of this species to other arthropods (Mazhawidza and Mvumi, 2017; Nyahangare et al., 2017). Compounds were tested at concentrations equivalent to those used by farmers highlighting a drawback of using plant chemicals as they may need to be used at high concentrations to be active. The cinnamoylagmatines were the most active and more active than the coumaroyl amides. While this suggests that the hydroxy moiety influences the activity, these differences may be accounted for also by concentrations as **1** and **2** were tested at concentrations lower than **3** and **4** being representative of their natural occurrence in the plant. The compounds activity, however, was only evident at similar efficacy to the positive control after 6 days so they are likely to be slower to effect kill than synthetic pesticides and therefore unlikely to be a perfect replacement for correctly used synthetics. However, where synthetics are not used or where synthetics are unavailable the aqueous extracts of this plant may provide an adequate alternative for farmers with no other options.

Agmatine derivatives are not known previously as insecticidal, however, phenyl propanoids are well documented as having biological activity against similar insects. For example, hydroxycinnamic acid esters of long chain alkanes were toxic to the sweet potato weevil *Cylas puncticollis* in artificial diets (Stevenson et al., 2009b) and explained the innate natural resistance in sweet potato to this pest weevil (Anyanga et al., 2013). The biological activity of the aqueous extract of *M. edulis* has been reported recently against two important pests of cabbage and *Brassica rapa Plutella xylostella* and *Brevicoryne brassicae* along with *Bobgunnia madagascariensis* (Mazhawidza and Mvumi, 2017). In *M. edulis* this activity might be explained by the agmatine derivatives identified in the present work because stachydrine and agmatine derivatives are polar molecules and are likely to be the only major components within the aqueous extract tested against the diamond backed moth and cabbage aphids. In the same study the biological activity of *B. madagascariensis* may similarly be due to the presence of water soluble saponins which are known to have biological activities also against bruchids (Stevenson et al., 2009a) but also to occur in this species (Stevenson et al., 2010).

*Maerua edulis* is used as a botanical insecticide by farmers in Southern Africa but the application of botanical insecticides is constrained by inefficient use and variation in active components that might be associated with provenance, location and time (Stevenson et al., 2017). Thus, a knowledge of the chemistry and the mechanisms of activity is required to optimize safety and use. This information could also help to identify elite plants for propagating the best materials that could support local use or commercialisation as an insecticidal product (Sarasan et al., 2011). Here we have identified the active components in the leaves that explain the biological activity against the target insects so further studies should determine the level of variation in the occurrence of these active components and what factors influence this, such as season, location or variety (Kamanula et al., 2017) as these can ultimately be important in determining whether a species has broad scale value as a botanical insecticide. The chemistry of plants can vary dramatically within the same species with significant consequences for users. For example, *Tephrosia vogelii* is a widely used pesticidal plant in Africa but occurs as two chemotypes (Stevenson et al., 2012) which have different biological activities against the same target insect from the present study, *C. maculatus*, (Belmain et al., 2012) so a knowledge of the chemistry can help understand why some users may have poor efficacy compared to others.

The potential for plant compounds to become commercial leads has been a topic of discussion in plant chemistry for decades with surprisingly few success stories (Isman, 2006). While interest had waned over the past 10–15 years, more recently, and particularly in light of regulatory changes to permitted chemistries in agriculture in Europe, interest is again growing with BRICS nations particularly leading the way (Isman, 2017). The agmatine derivatives from *M. edulis* in the present study were synthesized using a simple process with few steps that could be commercially viable. Further, bioactivity testing against a range of target species would help to determine if there was merit in pursuing the study of this plant as a broad-spectrum insecticide. In addition, understanding more about their biological activity against beneficial insects may also provide valuable information, while ensuring regulatory approval as a treatment with benign effects on the ecosystems. Recent work investigating botanical insecticide use suggests that plant compounds may not have the same efficacy compared to synthetic insecticides but may have reduced impacts against beneficial insects including pollinators and natural enemies of pests (Amoabeng et al., 2013; Mkenda et al., 2015) which might mean that they are better suited to sustainable intensification of agriculture.

A limiting factor for the commercialisation, however, particularly in Africa is the regulatory environment and despite the success of pyrethrum in some countries and other products from pesticidal plants in China and India, Africa is still behind the rest of the world in this field. Several factors need attention including data on efficacy and safety while the prohibitive cost of registration and an insufficiently developed conventional pesticides sector are also limitations (Sola et al., 2014).

## Author Contributions

PS, PG, IF, and SB designed the study. BM started the research which led to this and other studies. PS and IF carried out the chemical analysis with AB while PG carried out the bioassays. SB carried out the statistical analysis. PS, PG, and IF wrote the manuscript with significant editorial contributions from SB and BM. All authors were involved in writing the manuscript and gave final approval for publication.

## Conflict of Interest Statement

The authors declare that the research was conducted in the absence of any commercial or financial relationships that could be construed as a potential conflict of interest.
